# PReS-FINAL-2093: Can clinical response within 12 weeks in JIA-etanercept receivers predict the future clinical remission?

**DOI:** 10.1186/1546-0096-11-S2-P105

**Published:** 2013-12-05

**Authors:** P Pratsidou-Gertsi, E Verykouki, G Pardalos, M Trachana

**Affiliations:** 1 1st Dept of Pediatrics, Aristotle University, Hippokration General Hospital Thessaloniki, Greece; 2 Department of Hygiene and Epidemiology, Medical School, Aristotle University, Thessaloniki, Greece

## Introduction

Induction of inactive disease (ID) in Juvenile Idiopathic Arthritis (JIA) is the main target of all current regimes, as it will maintain physical ability and hinder disease associated-damage. Early predictive markers for the future achievement of ID in patients under expensive therapies are therefore urgently needed in the era of financial recession. Our previous published experience reported that ≥ 50% of the JIA patients achieved Clinical Remission (CR) following ≥12-month (CR12) Etanercept (ETN) administration.

## Objectives

To assess the early ID following 12 weeks-ETN treatment (eID) and explore the eID's prognostic value for the CR12.

## Methods

JIA patients treated with ETN for ≥12 months during the last decade were enrolled in the study. Baseline, 12 week and 12 month disease assessments were built on: a) the Juvenile Arthritis Disease Activity Score (JADAS) and b) the fulfilment of Minimal Disease Activity (MDA) and Inactive Disease (ID) definitions, as well as CR according to Wallace's criteria.

## Results

52 JIA patients (F:40) with a median age of 8.8 years at first ETN dose, mainly with a polyarticular course (47) and a median disease duration of 4.04 years were studied. eID was achieved in 26/52 (50%) of the patients and CR12 in 28/52 (53.84%). The following factors were found to be independent of eID: disease duration (p = 0.23), JIA classification and course (p = 0.51, p = 0.58, respectively), age at first ETN dose (p = 0.26), presence of ANA or RF (p = 0.43, p = 0.37, respectively) and JADAS at baseline (p = 0.12). Interestingly, the 3 levels of disease activity (persistent activity, MDA and ID) 3 months post-treatment were associated with the 12 month post-treatment JADAS and CR (p = 0.001 and p ≤ 0.001, respectively).

## Conclusion

ETN achieved Inactive Disease in 50% of the patients within a-12 week administration and CR in the 54% of all patients after 12 months of therapy. These findings concord with recent publications. However, neither demographic nor JIA characteristics were found to be predictors of eID. Finally, the beneficial 3 month impact of ETN was evidenced by its association with CR12. The disease regression proves the drug's early efficacy in JIA patients and supports the clinician's management decision regarding the application of expensive medication in the era of financial recession.

## Disclosure of interest

P. Pratsidou-Gertsi Grant/Research Support from: Abbvie, Novartis, Pfizer, Consultant for: Novartis, E. Verykouki: None declared, G. Pardalos Grant/Research Support from: Novartis, M. Trachana Grant/Research Support from: Abbvie, Novartis, Pfizer, Consultant for: Novartis.

